# Comparison of marker types and map assumptions using Markov chain Monte Carlo-based linkage analysis of COGA data

**DOI:** 10.1186/1471-2156-6-S1-S11

**Published:** 2005-12-30

**Authors:** Weiva Sieh, Saonli Basu, Audrey Q Fu, Joseph H Rothstein, Paul A Scheet, William CL Stewart, Yun J Sung, Elizabeth A Thompson, Ellen M Wijsman

**Affiliations:** 1 Division of Medical Genetics, Department of Medicine, University of Washington, Seattle, Washington, 98195, USA; 2 Department of Statistics, University of Washington, Seattle, Washington, 98195, USA; 3 Department of Biostatistics, University of Washington, Seattle, Washington, 98195, USA

## Abstract

We performed multipoint linkage analysis of the electrophysiological trait ECB21 on chromosome 4 in the full pedigrees provided by the Collaborative Study on the Genetics of Alcoholism (COGA). Three Markov chain Monte Carlo (MCMC)-based approaches were applied to the provided and re-estimated genetic maps and to five different marker panels consisting of microsatellite (STRP) and/or SNP markers at various densities. We found evidence of linkage near the GABRB1 STRP using all methods, maps, and marker panels. Difficulties encountered with SNP panels included convergence problems and demanding computations.

## Background

Our aims were to investigate 1) the utility of single-nucleotide polymorphisms (SNPs) versus microsatellites (STRPs), and 2) the impact of map assumptions on linkage analysis. We chose to focus our analyses on the COGA ECB21 trait and chromosome 4 because previous studies [1,2] had reported significant evidence for linkage of the electroencephalogram (EEG) beta wave to chromosome 4. Multipoint linkage analysis of the full pedigree structures was performed by using MCMC techniques to implement allele-sharing, parametric LOD score, and Bayesian analysis approaches.

## Methods

### Trait definition and segregation analyses

A multivariate polygenic model was used to obtain maximum likelihood estimates of the heritabilities and genetic correlations of ECB21 and 12 other EEG measurements [3]. On the basis of the results, ECB21 and TTTH3 were selected for further study. Early analyses of TTTH3 showed little evidence of linkage to chromosome 4, so subsequent analyses focused only on ECB21. Oligogenic segregation analysis [4] of ECB21, adjusting for age and gender, revealed two quantitative trait locus (QTL) models. The model with the highest posterior probability provided stronger evidence of linkage to chromosome 4 and was used in subsequent parametric LOD score analysis of the quantitative trait, ECB21_Q, preadjusted for age and gender. We created a dichotomous trait, ECB21_D, by defining ECB21_Q ≥ 3 as 'affected'. This cutpoint maximized the difference between the penetrances of the high- versus low-risk genotypes based on the estimated genotype effects from the most likely QTL model.

### Map construction

All 275 Illumina SNPs on chromosome 4 and 550 consecutive Affymetrix SNPs spanning STRPs 2–12 on chromosome 4 were selected. Among SNPs with identical meiotic map positions, the SNP with the largest minor allele frequency was retained, leaving a relatively sparse panel of 140 Illumina SNPs with an average spacing of ~1.5 cM (ILMN_1.5) and a dense panel of 476 Affymetrix SNPs with an average spacing of 0.3 cM (AFFY_0.3) for further analysis. A subset of 97 Affymetrix SNPs (AFFY_1.5) was selected by requiring an empirically determined minimum distance of 1.1 cM between SNPs, starting from the first SNP, to achieve a similar average density as ILMN_1.5. SNPs were interpolated onto the COGA STRP map by pegging the two flanking SNPs to each STRP and interpolating the intervening SNPs based upon the proportional distances in the corresponding intervals on the COGA and provided SNP maps.

Genetic maps were re-estimated from the COGA data using a hybrid algorithm, based on MCMC-EM (expectation maximization) and stochastic approximation for STRPs and MCMC-EM for SNPs, to find the maximum likelihood estimates of the recombination fractions. Sex-averaged and sex-specific maps were re-estimated using all 17 STRPs on chromosome 4, and a sex-averaged map was estimated using STRPs 2–12 plus AFFY_0.3. Haldane map distances were used in all analyses and figures.

### Linkage analyses

Linkage analyses of the ECB21 traits on chromosome 4 used three MCMC-based methods from the MORGAN and Loki software packages [5]. First, a MORGAN IBD-scoring program (lm_ibdtest) was used to analyze ECB21_D. This program obtains MCMC estimates of the allele-sharing statistic S_pairs _[6] and determines significance levels with a permutation test rather than relying upon normality assumptions. Second, a MORGAN parametric LOD score program (lm_markers) was used to analyze ECB21_D (not shown) and ECB21_Q using parameters from the segregation model for ECB21_Q and the associated penetrances and allele frequencies for ECB21_D. Third, an oligogenic linkage analysis approach (Loki) was used to analyze ECB21_Q; results are expressed as Bayes factors, or the posterior:prior odds that a QTL exists in a given 2cM region. A 50:50 ratio of locus to meiosis block Gibbs sampling [7] was used in all analyses. Initial starting configurations were obtained by using the locus sampler independently on each locus. We performed single-marker analyses with each of the 17 STRPs on chromosome 4. Multipoint analyses used five marker panels: 17 STRPs; AFFY_0.3; STRPs 2–12 plus AFFY_0.3; ILMN_1.5; and AFFY_1.5.

To evaluate the effects of the real chromosome 4 STRP data and provided map on type I error, 1,000 replicates of an unlinked quantitative trait, based on the ECB21_Q model, were simulated on the COGA pedigrees. The simulated trait was then dichotomized using the same cut point as for ECB21_D. For comparison, true null datasets were created by pairing each of the 1,000 unlinked trait replicates with a single set of unlinked markers, simulated based on the chromosome 4 STRP allele frequencies and map. S_pairs _was computed at each marker in each replicate using lm_ibdtest.

## Results

### Trait definition and segregation analyses

The polygenic analysis estimated a narrow-sense heritability of 0.61 for ECB21_Q and genetic correlation of 0.47 between ECB21 and TTTH3. Oligogenic segregation analysis of ECB21_Q indicated the existence of at least one QTL. The estimated parameters for the most likely QTL model were: frequency of 0.411 for the minor allele "A", genotype means μ_aa _= -1.22, μ_Aa_= -1.14, μ_AA _= 5.79, and residual variance of 22.0. Penetrances for ECB21_D were 19%, 19%, and 73% for the aa, Aa, and AA genotypes, respectively.

### Map construction

**Figure 1:**
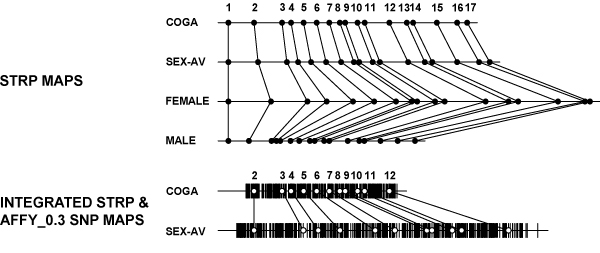
**Genetic maps of chromosome 4. **Genetic distances for the 17 chromosome 4 STRPs (●) on the COGA map or the re-estimated sex-averaged, female, and male maps. Integrated maps for the STRPs (○) and AFFY_0.3 SNPs (|) based on interpolation of SNPs onto the COGA map or map estimation from the data.

The re-estimated maps based on STRPs were similar to those provided and published, but there was substantial map inflation when SNPs were included (Figure 1). The sex-averaged distance between STRPs 1–17 on chromosome 4 was slightly longer on the re-estimated map (255 cM) compared to the COGA map (233 cM) converted to Haldane distances. Consistent with published maps [8], the estimated female map (351 cM) was much longer than the male map (183 cM), especially near STRP 4. Map distances estimated using the joint STRP and AFFY_0.3 panel were substantially inflated compared to the COGA map: 248 cM versus 132 cM between STRPs 2–12, respectively. Therefore, maps based on interpolation of SNPs onto the COGA map were used for all SNP analyses.

### Linkage analyses

**Figure 2:**
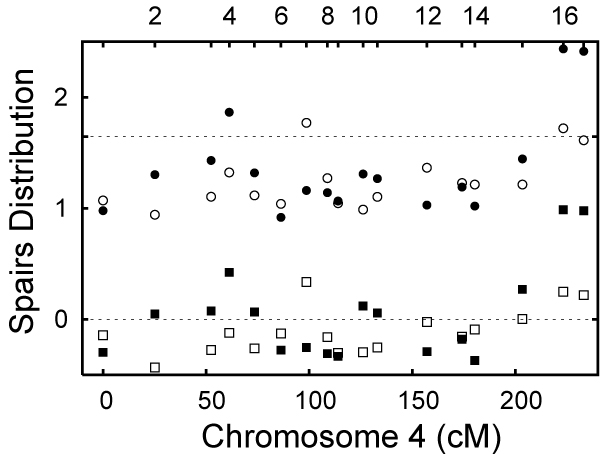
**Simulated unlinked trait. **S_pairs _distribution for 1,000 datasets with a simulated unlinked trait and the real STRP data (solid symbols) and 1,000 true null datasets (open symbols). The 50^th ^(squares) and 95^th ^(circles) percentiles of the S_pairs _distributions at each STRP are shown. Numbers and tick markers on the top axis denote STRPs and their positions. Dotted lines indicate the 50^th ^and 95^th ^percentiles assuming a normally distributed score.

We observed a strong linkage signal near STRP 4 that was insensitive to the STRP map estimate. Whereas the COGA and re-estimated sex-averaged maps provided similar linkage results, small differences resulted from use of the estimated sex-specific map. The largest change in the permutation-based *p*-value for S_pairs _was an increase from *p *= 0.007 with the COGA map to *p *= 0.023 with the sex-specific map for ECB21_D at STRP 10. The empirical distribution of S_pairs_, based upon 1,000 replicates of a simulated unlinked trait and the real chromosome 4 STRPs, showed an excess of allele-sharing at STRPs 4, 16, and 17, whereas little excess sharing was observed with the true null replicates (Figure 2). Inflation of type I error rates using the real genotype data persisted when maps re-estimated from the data were used (not shown).

**Figure 3:**
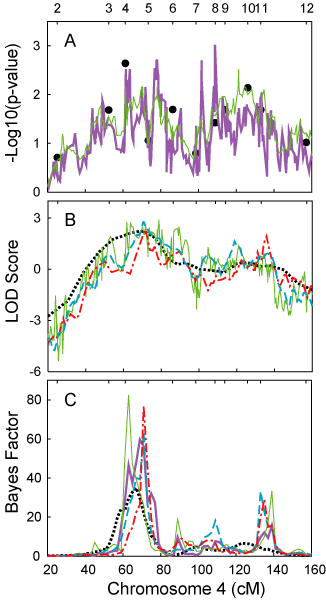
**Linkage analyses of ECB21 on chromosome 4. **Linkage results for three MCMC approaches (A-C) and 5 marker panels: STRPs only (black dots in A or dotted line in B-C), AFFY_0.3 (thick purple line), STRPs plus AFFY_0.3 (thin green line), ILMN_1.5 (red dashes and dots), and AFFY_1.5 (blue dashes). (A) Negative log_10 _of the *p*-values for S_pairs _for ECB21_D. (B) LOD scores for ECB21_Q. (C) Bayes factors for ECB21_Q. Numbers and tick markers on the top axis denote STRPs and their positions.

Multipoint STRP scans with three different MCMC-based methods all showed evidence of linkage of the ECB21 traits to chromosome 4 (Figure 3). The strongest signal was near STRP 4, with a weaker positive signal near STRP 10 for all analysis methods. There was no evidence of heterogeneity among the 143 COGA families using LOD scores for individual families in a heterogeneity test. Replicate runs gave similar results: for example, the standard deviation of the maximum LOD score was 0.2 in five runs with lm_markers. Results for single-STRP analyses were similar to multipoint results near marker 4, and in some cases provided stronger evidence of linkage near markers 10–11 than did the multipoint analyses.

**Figure 4:**
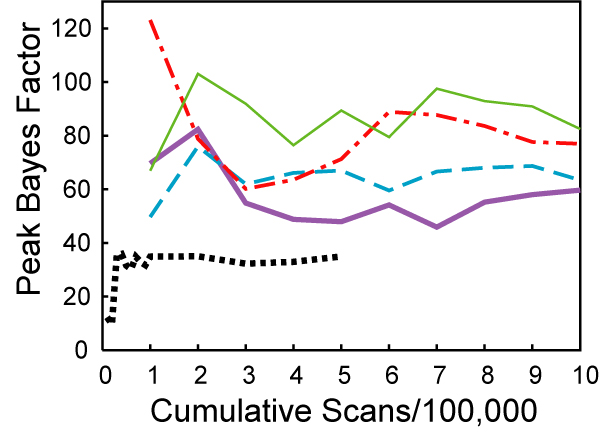
**Convergence of Bayes factor. **Analyses of ECB21_Q on chromosome 4 using 17 STRPs (black dotted line), 476 AFFY_0.3 SNPs (thick purple line), 487 combined STRPs and AFFY_0.3 SNPs (thin green line), 140 ILMN_1.5 SNPs (red dashes and dots), and 97 AFFY_1.5 SNPs (blue dashes). Bayes factors for the final chromosome position of the strongest peak were estimated at intermediate points during the run.

MCMC multipoint analyses with STRPs versus SNPs yielded similar results in the chromosome 4 60–80 cM region, but also gave important differences. AFFY_0.3 results were noisy compared to STRP results (Figure 3A–B), and numerous suggestive peaks across broad regions created difficulties in localizing the signal(s). The sparse SNP panels produced smoother LOD score curves than the dense panel and narrower 1-LOD support intervals than the STRPs (Figure 3B). The magnitude of the peak LOD score was similar for all marker panels despite differences in density and marker type. Small secondary peaks were observed with the SNPs that were not consistent across panels. These weak signals could be the result of linkage disequilibrium, undetected genotype error, and/or MCMC mixing problems. Oligogenic linkage analyses with SNP panels (Figure 3C) showed evidence of poor mixing: whereas the Bayes factor at the final location of the strongest peak converged after 100,000 iterations for the STRPs, convergence was still not reached after one million iterations with any of the SNP panels (Figure 4). The dense SNP panel did not provide more evidence for linkage compared to the sparse SNP panels, but SNPs may yield larger maximum Bayes factors and narrower peaks than STRPs. These results must be interpreted with caution due to poor mixing of the MCMC sampler in the SNP analyses. The computational demands of SNP analyses were substantially greater than for STRPs: the CPU time for 17 STRPs vs. 476 SNPs was ~9 min vs. ~2.5 hr for 2,000 MCMC scans with lm_ibdtest, and ~15 min vs. ~5 hr for 4,000 scans with lm_markers on a Xeon 3.06 GHz processor; and ~1 day vs. ~2 weeks for 1 million scans with Loki on a Xeon 2.66 GHz processor.

## Conclusion

Three different MCMC-based multipoint methods gave evidence in the COGA STRP data for linkage of ECB21 to STRP 4 on chromosome 4. We also found weaker evidence of linkage near STRP 10. Comparison of sex-averaged and sex-specific STRP maps suggested that results may be robust to map-misspecification in the presence of strong evidence for linkage. However, the investigation of map assumptions may be important in elucidating weak linkage signals, especially in chromosomal regions with substantial male-female map differences. Map estimation using SNP data led to substantial expansion of genetic distances compared to maps estimated from STRP data, suggesting possible undetected SNP genotype errors or effects of linkage disequilibrium. Our analyses of simulated null datasets with an unlinked trait and real STRP data indicated that some regions of chromosome 4, including STRP 4, may be prone to false-positive linkage signals, and that this tendency persists even using maps estimated from the data. Possible explanations for false-positive results include genotype error or allele frequency misspecification.

Multipoint analyses using STRPs, SNPs, or a combination of STRPs and SNPs yielded comparable evidence of linkage to the chromosome 4 region with the strongest signal. The signal strength was not greater for the dense versus sparse SNP panels. Furthermore, localization and interpretation of linkage signals for the dense SNP panel were complicated by noisy results, which could reflect MCMC mixing problems and/or genotype error. Multipoint analyses using sparse SNP panels produced smoother LOD score curves than the dense SNPs. These results suggest that increasing the density of SNP panels beyond an average spacing of 1.5 cM does not substantially increase the evidence for linkage in the COGA dataset, which consists of moderate-size pedigrees with relatively complete genotype data. Additional studies will be needed to determine the optimal density for SNP panels in other datasets. Our analyses with current MCMC approaches indicate that, while useable with dense SNPs in limited chromosome regions with medium-size pedigrees, long runs are needed to produce stable linkage analysis results. Run times may prohibit the use of dense SNP panels for whole-genome scans with current MCMC analysis programs. MCMC-based methods are among the best tools now available for the analysis of large pedigrees, numerous markers, and complex traits. Further development of these methods in order to accommodate dense SNP panels in the context of large pedigrees would be of value.

## Abbreviations

COGA: Collaborative Study on the Genetics of Alcoholism

EEG: Electroencephalogram

EM: Expectation maximization

GAW: Genetic Analysis Workshop

MCMC: Markov chain Monte Carlo

QTL: Quantitative trait locus

SNP: Single-nucleotide polymorphism

STRP: Short tandem repeat polymorphism
